# Molecular mechanisms of hydrogen sulfide against uremic accelerated atherosclerosis through cPKCβII/Akt signal pathway

**DOI:** 10.1186/s12882-019-1550-4

**Published:** 2019-09-14

**Authors:** Ruifang Xiong, Xiangxue Lu, Jinghong Song, Han Li, Shixiang Wang

**Affiliations:** Department of Blood Purification, Beijing Chao-Yang Hospital, Capital Medical University, No. 8 Gongti South Road, Chaoyang District, Beijing, 100020 China

**Keywords:** Uremia accelerated atherosclerosis, ApoE^−/−^ mice, Hydrogen sulfide, cPKCβII/Akt signaling pathway, Endothelial nitric oxide synthase

## Abstract

**Background:**

Cardiovascular disease is the most common complication and leading cause of death in maintenance hemodialysis patients. The protection mechanism of hydrogen sulfide (H_2_S) and the specific role of conventional protein kinase C βII (cPKCβII)/Akt signaling pathway in the formation of atherosclerosis is still controversial.

**Methods:**

8-week-old male ApoE^−/−^ mice were treated with 5/6 nephrectomy and high-fat diet to make uremia accelerated atherosclerosis (UAAS) model. Mice were divided into normal control group (control group), sham operation group (sham group), UAAS group, L-cysteine group (UAAS+L-cys group), sodium hydrosulfide group (UAAS+NaHS group), and propargylglycine group (UAAS+PPG group). Western blot was used to detect cPKCβII activation, Akt phosphorylation and endothelial nitric oxide synthase (eNOS) expression in mice aorta.

**Results:**

The membrane translocation of cPKCβII in UAAS group was higher than sham group, and L-cys or NaHS injection could suppress the membrane translocation, but PPG treatment resulted in more membrane translocation of cPKCβII (*P* < 0.05, *n* = 6 per group). Akt phosphorylation and the eNOS expression in UAAS group was lower than sham group, and L-cys or NaHS injection could suppress the degradation of Akt phosphorylation and the eNOS expression, but PPG treatment resulted in more decrease in the Akt phosphorylation and the eNOS expression (*P* < 0.05, *n* = 6 per group).

**Conclusion:**

Endogenous cystathionine-γ-lyase (CSE)/H_2_S system protected against the formation of UAAS via cPKCβII/Akt signal pathway. The imbalance of CSE/H_2_S system may participate in the formation of UAAS by affecting the expression of downstream molecule eNOS, which may be mediated by cPKCβII/Akt signaling pathway.

## Background

Cardiovascular disease is the most common complication and leading cause of death in maintenance hemodialysis patients [1]. The incidence of cardiovascular disease in chronic kidney disease (CKD) patients is 80%, and the risk of death from cardiovascular events in hemodialysis patients is 10 to 20 times that of the general population [2]. The episode age of atherosclerosis is advanced, and vascular lesions progress faster in patients with end-stage renal disease, which is known as uremia accelerated atherosclerosis (UAAS), the age of onset is generally 30 to 40 [3]. As atherosclerosis is the main predictor of death of cardiovascular disease in hemodialysis patients, studies on occurrence and progression of atherosclerosis in uremic patients are of great significance for the prevention and treatment of cardiovascular diseases [4].

The damage of arterial endothelium is recognized as initial factor for atherosclerosis and plays an important role in the occurrence and development of cardiovascular diseases [5]. Our group found that there was a relationship between endogenous cystathionine-γ-lyase/hydrogen sulfide (CSE/H_2_S) system and the risk of cardiovascular disease in maintenance hemodialysis patients [6]. Abnormal metabolism of endogenous H_2_S accelerated the progression of atherosclerosis in patients with diabetic nephropathy [7]. We further found that uremia accelerated the progression of atherosclerosis in ApoE^−/−^ mice and exogenous H_2_S can inhibit the progression of atherosclerosis in uremia ApoE^−/−^ mice. Protein kinase C (PKC) is an important signal transduction molecule in cells [8]. Akt is one of the most versatile kinases in the human kinome and is critical regulator of human physiology that controls diverse cellular functions [9]. It was shown that the level of Akt phosphorylation was changed during the development and progression of atherosclerosis [10]. We found that H_2_S level in patients with maintenance hemodialysis was lower than that in the normal population, with increased cPKCβII activation and decreased Akt phosphorylation [6]. Other studies have shown that cPKCβII inhibitors can slow down the progression of cardiovascular disease [11]. These studies suggest that abnormal H_2_S metabolism was involved in the progression of uremia with cardiovascular disease and may be achieved through PKC signaling pathways. H_2_S and nitric oxide (NO) have a synergistic effect on vasodilation. Exogenous H_2_S can enhance the relaxation of NO on blood vessels [12]. The activation of cPKCβII is increased in aortic endothelial cells of diabetic patients, and PKC inhibitors improved insulin-mediated endothelial nitric oxide synthase (eNOS) activation in patients with diabetes mellitus, which suggested that H_2_S can regulate the production of eNOS through a certain pathway and participate in the regulation of endothelial function [13].

Large numbers of studies have shown that H_2_S has a protective role in the formation of atherosclerosis, [14, 15] but its protection mechanism is still not clear. cPKCβII/Akt signaling pathway is widely involved in the occurrence and development of cardiovascular diseases such as atherosclerosis, myocardial ischemia-reperfusion injury, and heart failure, but the specific role of cPKCβII/Akt signaling pathway in the formation of atherosclerosis remains controversial. The aim of this study was to identify the possible molecular mechanism of the CSE/H_2_S system and cPKCβII/Akt signaling pathway on atherosclerosis development in UAAS mice.

## Methods

### Materials

BCA protein quantification kit (Applygen), sodium hydrosulfide (Sigma-aldrich), L-cysteine (Sinopharm), propargylglycine (Aladdin), Anti-eNOS Rabbit Antibody (Santa Cruz), Anti-p-Akt Mouse Antibody (Santa Cruz), Anti-t-Akt Mouse Antibody (Santa Cruz), Anti-cPKCβII Rabbit Antibody (Santa Cruz), HRP-labeled Goat Anti-Mouse IgG (Applygen), HRP-labeled Goat Anti-Rabbit IgG (Applygen), β-actin, Mouse mAb (Applygen), Multi-functional enzyme immunoassay (Thermo), Gel imager (Bio-Rad), Membrane and cytosol protein extraction kit (Beyotime).

### Animals and UAAS model preparation

Male ApoE^−/−^ mice were purchased from Beijing Vital River Laboratory Animal Technology Co., Ltd. (License No. SCXK (Beijing) 2016–0006). Mice were reared in the clean level laboratory with temperature of 18–25 °C and relative humidity of 35–50%, sufficient oxygen was provided, 4 mice per cage. Our operations are in accordance with the NIH Guide for the Care and Use of Laboratory Animals. All procedures with animals in this study were approved by the Institutional Animal Care and Use Committee of Beijing Chao-Yang Hospital, Capital Medical University.

Mice were divided into control group, sham group, UAAS group, L-cysteine group (UAAS+L-cys group), sodium hydrosulfide group (UAAS+NaHS group), and propargylglycine group (UAAS+PPG group) (*n* = 6 for each group). NaHS (a donor of H_2_S) and L-cys (a precursor of H_2_S generation) were used as a source of H_2_S, and PPG is a selective inhibitor of CSE, which is an important H_2_S-synthesizing enzyme [16]. At 8 weeks of age, the mice were anesthetized with sodium pentobarbital by intraperitoneal injection (0.06 g/kg). A longitudinal incision of approximately 1 cm was made at the lower lateral of the left costovertebral angle of the mice. Then the left kidney was exposed and the renal capsule was isolated. A total of 2/3 of the kidney tissue was removed from the upper and lower poles of the left kidney, then a gelatin sponge was used to compress and stop bleeding. After that, the muscles and skin were sutured. After 2 weeks, the right kidney was exposed in the same way, and the right renal pedicle was ligated. After confirming complete ligation, the right kidney was excised [17]. In the sham group, the kidneys were only exposed during the two operations and no surgical resection was performed. All mice were fed a high-fat diet (Cat. No.: D12108C). After the operation, L-cys(50 mg/kg/d), NaHS(56 μmol/kg/d) and PPG(37.5 mg/kg/d) were intraperitoneally injected in UAAS+L-cys group, UAAS+NaHS group, and UAAS+PPG group respectively for 6 weeks. After the intraperitoneally injection for 6 weeks, mice were euthanized via cervical dislocation according to the American Veterinary Medical Association and aortas were dissected and separated from fatty tissue, rinsed with normal saline and stored in a − 80 °C freezer.

### Extraction of cytosolic and membrane proteins

The membrane protein extraction reagent A was added to the aortic tissue debris (PMSF was added 2 min earlier, make the final PMSF concentration of 1 mM), homogenized, sonicated, vortex reconstituted, then centrifuged at 700 g for 10 min at 4 °C, the supernatant was collected as cytoplasmic protein. The membrane protein extraction reagent B was added to the precipitate (PMSF was added 2 min earlier, make the final PMSF concentration of 1 mM), homogenized, sonicated, vortex reconstituted, and centrifuged at 14000 g for 5 min at 4 °C, then the membrane protein was collected and stored at − 80 °C for use.

### Extraction of total protein

100uL of Pre-chilled RIPA lysis buffer was added to the cleaved aortic tissue, homogenized, sonicated, vortex reconstituted, then centrifuged at 12000 g for 10 min at 4 °C, the supernatant was collected as total protein, stored at − 80 °C for use.

### Western blot

Protein levels of cPKCβII of cytoplasmic and membrane protein, cPKCβII, Akt, phosphorylated-Akt (p-Akt), eNOS of total protein in mice aorta were analyzed by Western blot. The concentration of cytoplasmic, membrane protein and tissue total protein were detected by BCA method. 20 μg of each sample were electrophoresed on a 10% SDS-polyacrylamide gel. The protein was electrophoresed at a voltage of 80 V, and was increased to 120 V when the protein runs to the separation gel. After completion of electrophoresis, the protein was transferred from SDS-PAGE to polyvinylidene fluoride film (PVDF) at 12 V for 100 min. The PVDF membranes were blocked with 10% skim milk which was formulated in Tris-HCI buffered saline solution (TBST) for 60 min, then the membrane was washed for 3 times with TBST for 10 min each. The membrane was probed with the following antibodies: β-actin (goat anti-mouse, 1:5000 dilution, Applygen), cPKCβII (goat anti-rabbit, 1:5000 dilution, Santa Cruz), p-Akt (goat anti-rabbit, 1:5000 dilution, Santa Cruz), t-Akt (goat anti-rabbit, 1:5000 dilution, Santa Cruz), eNOS (goat anti-rabbit, 1:5000 dilution, Santa Cruz). The membrane was incubated overnight at 4 °C, after 3 times with TBST for 10 min each, membranes were incubated with HRP-labeled Goat Anti-Mouse IgG (1:5000 dilution, Applygen) or HRP-labeled Goat Anti-Rabbit IgG (1:5000 dilution, Applygen). The membranes were placed on a bleaching shaker for 1 h at room temperature. A, B color mixture (1:1) was dropped on membrane and immediately placed in a gel imager (Bio-Rad, USA) until the best band was appeared.

### Statistical analysis of data

Western blot results were semi-quantitatively analyzed using Image Lab software, SPSS22.0 statistical software (SPSS for Windows, Version 22.0, SPSS, USA) was used to statistically analyze the experimental data. Results were expressed as mean ± standard deviation ($\overline{\mathrm{x}}$ ±s). One-Way ANOVA was used between groups and LSD method was used each group. *P* < 0.05 was considered statistical significance. GraphPad Prism 6.0 statistical plotting software was used to plot data graphs.

## Results

### CSE/H_2_S regulates activation of cPKCβII against formation of UAAS in mouse aorta (Fig. 1)

Aortic cytosolic and membrane protein was extracted and membrane translocation of cPKCβII was detected. The membrane translocation of cPKCβII was of no significant difference between sham group and control group (*P* = 0.345, *n* = 6); The membrane translocation of cPKCβII in UAAS group was higher than sham group (*P* = 0.015, n = 6), meanwhile, compared with UAAS group, L-cys (*P* = 0.010, n = 6) or NaHS (*P* = 0.012, n = 6) injection could suppress the membrane translocation, but PPG treatment (*P* = 0.031, n = 6) resulted in more membrane translocation of cPKCβII with differences of statistical significance.

**Fig. 1:**
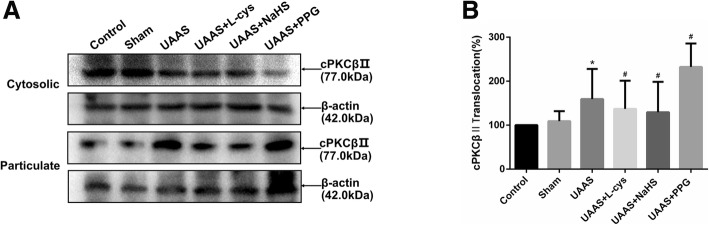
Effects of CSE/H_2_S system on the membrane translocation of cPKCβII in mouse aorta. The membrane translocation of cPKCβII in control, sham, UAAS, UAAS+L-cys, UAAS+NaHS and UAAS+PPG group. (**a**) The protein contents in cytosolic and particulate fraction of mouse aorta were tested by Western blot; (**b**) Quantitative analysis showed that cPKCβII membrane translocation in UAAS group increased significantly compared with sham group (^*^P < 0.05 vs. sham group, *n* = 6 per group). L-cys or NaHS injection could suppress the membrane translocation, but PPG treatment resulted in more membrane translocation of cPKCβII (^#^*P* < 0.05 vs. UAAS group, *n* = 6 per group)

### CSE/H_2_S system has no effect on total protein cPKCβII expression in mouse aorta (Fig. 2)

Total protein of mouse aorta was extracted and the expression of cPKCβII was compared between groups, there were no significant difference in cPKCβII expression between each group.

**Fig. 2:**
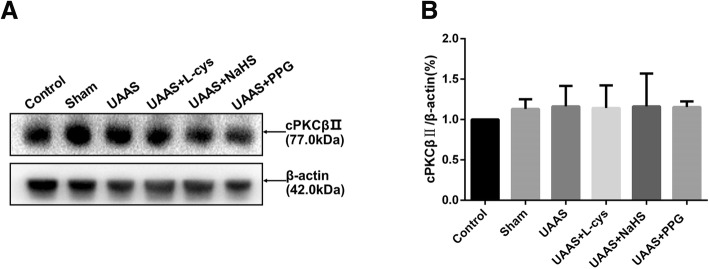
Effects of CSE/H_2_S system on the total expression level of cPKCβII in mouse aorta. The total expression level of cPKCβII in control, sham, UAAS, UAAS+L-cys, UAAS+NaHS and UAAS+PPG group. (**a**) The protein contents in mouse aorta were tested by Western blot; (**b**) Quantitative analysis showed there were no significant difference in the expression of total cPKCβII in each group

### CSE/H_2_S regulates Akt phosphorylation against UAAS formation in mouse aorta (Fig. 3)

Total mouse aortic protein was extracted and Akt phosphorylation was detected. Akt phosphorylation was of no significant difference between sham group and control group (*P* = 0.362, *n* = 6); Akt phosphorylation in UAAS group was lower than sham group (*P* = 0.000001, n = 6), meanwhile, compared with UAAS group, L-cys (*P* = 0.000054, n = 6) or NaHS (*P* = 0.000010, n = 6) injection could suppress the degradation of Akt phosphorylation, but PPG treatment (*P* = 0.005836, *n* = 6) resulted in more decrease in the Akt phosphorylation with differences of statistical significance.

**Fig. 3:**
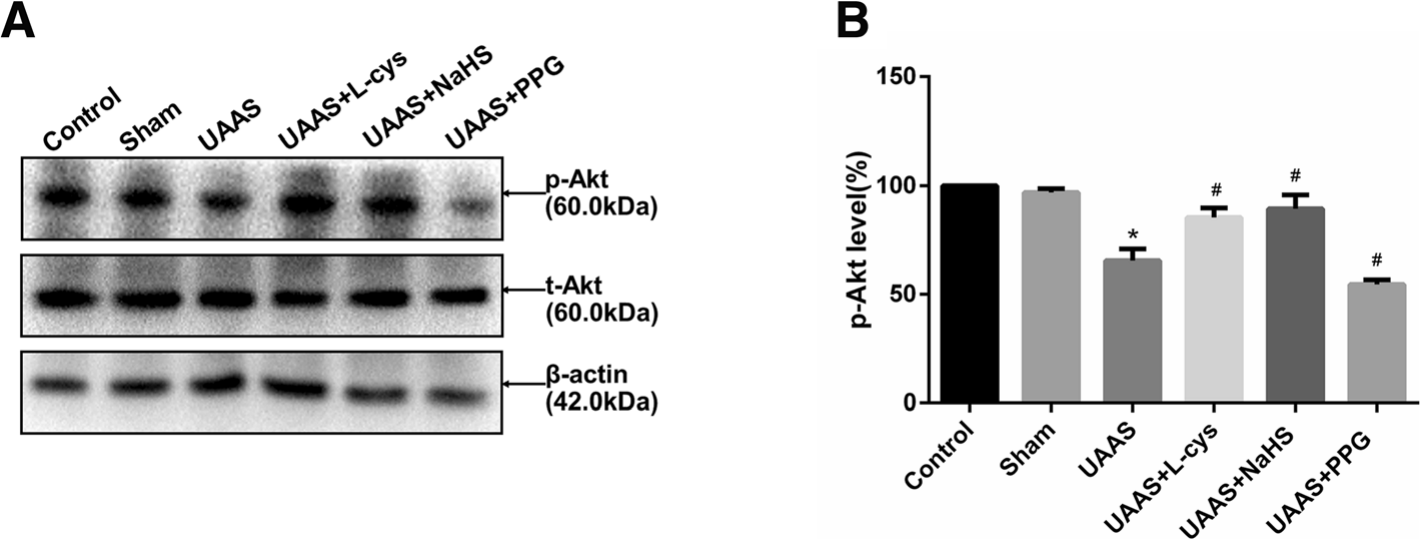
Effects of CSE/H_2_S system on the phosphorylation level of Akt in mouse aorta. The phosphorylation level of Akt in control, sham, UAAS, UAAS+L-cys, UAAS+NaHS and UAAS+PPG group. (**a**) The protein contents in mouse aorta were tested by Western blot; (**b**) Quantitative analysis showed that Akt phosphorylation in UAAS group decreased significantly compared with sham group (^*^P < 0.05 vs. sham group, *n* = 6 per group). L-cys or NaHS injection could suppress the degradation of Akt phosphorylation, but PPG treatment resulted in more decrease in the Akt phosphorylation (^#^*P* < 0.05 vs. UAAS group, *n* = 6 per group)

### CSE/H_2_S system regulates eNOS expression in mouse aorta against UAAS formation in mice aorta (Fig. 4)

Total mouse aortic protein was extracted and expression of eNOS protein was detected. Expression of eNOS was of no significant difference between sham group and control group (*P* = 0.345, *n* = 6); The expression of eNOS in UAAS group was lower than sham group (*P* = 0.034, n = 6), meanwhile, compared with UAAS group, L-cys (*P* = 0.028, n = 6) or NaHS (*P* = 0.012, n = 6) injection could suppress the degradation of eNOS expression, but PPG treatment (*P* = 0.011, n = 6) resulted in more decrease in the eNOS expression with the difference of statistical significance.

**Fig. 4:**
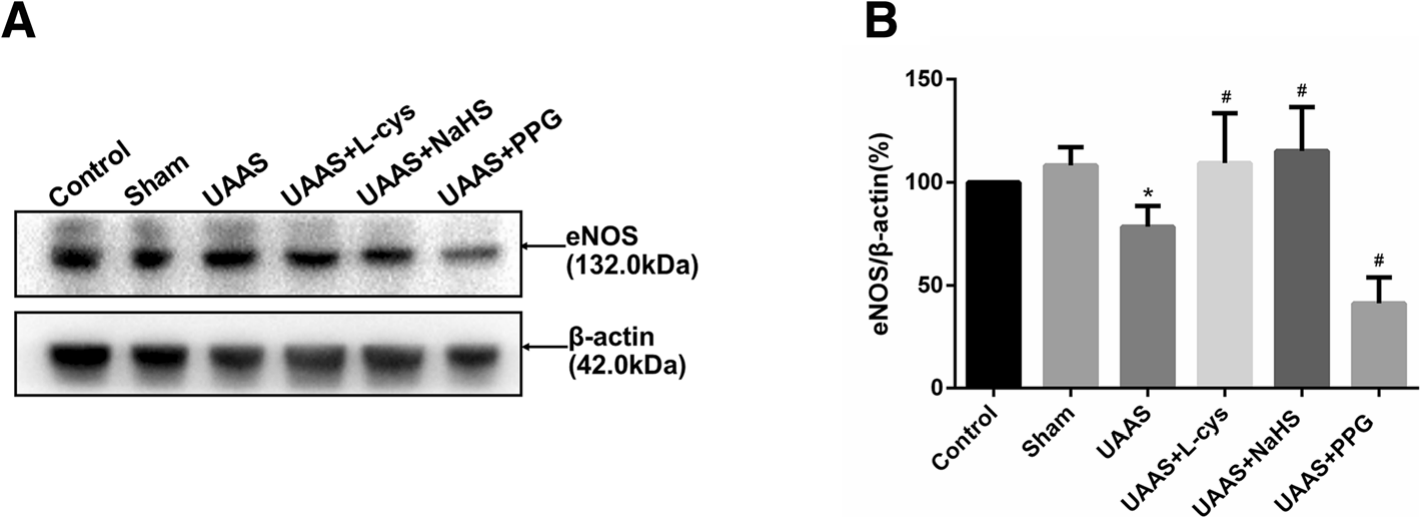
Effects of CSE/H_2_S system on the expression of eNOS in mouse aorta. The expression of eNOS in control, sham, UAAS, UAAS+L-cys, UAAS+NaHS and UAAS+PPG group. (**a**) The protein contents in mouse aorta were tested by Western blot; (**b**) Quantitative analysis showed that eNOS expression in UAAS group decreased significantly compared with sham group (^*^P < 0.05 vs. sham group, *n* = 6 per group). L-cys or NaHS injection could suppress the degradation of eNOS expression, but PPG treatment resulted in more decrease in the eNOS expression (^#^*P* < 0.05 vs. UAAS group, *n* = 6 per group)

## Discussion

Patients with end-stage renal disease have increased cardiovascular morbidity and mortality [1]. Atherosclerosis is the main predictor of death of cardiovascular diseases in hemodialysis patients, and arterial endothelium damage is recognized as initial factor for atherosclerosis.

In mammals, H_2_S is mainly produced through both enzymatic and nonenzymatic pathways. In enzyme synthesis pathway, endogenous H_2_S is mainly produced by the metabolism of cysteine. Currently, there are five enzymes involved in the formation of H_2_S, namely cystathionine-β-synthase (CBS), cystathionine-γ-lyase (CSE), 3-mercaptopyruvate sulfurtransferase (3-MST), cysteine aminotransferase (CAT) and D- D-amino acid oxidase (DAO) [18]. Abnormal metabolism of endogenous H_2_S accelerated the progression of atherosclerosis in patients with diabetic nephropathy [7]. Others showed that the occurrence of atherosclerosis and hypertension is associated with CSE/H_2_S disorders in chronic hemodialysis patients [19]. These findings indicate that endogenous H_2_S has a certain inhibitory effect on the progression of atherosclerosis.

PKC is an important signal transduction molecule in cells. Currently PKC are divided into 3 subtypes, traditional PKC (α, βI, βII, γ), novel PKC (δ, ε, η, θ), atypical PKC (ζ, λ ) [8]. Several studies have shown that cPKCβII plays very important role in the development of atherosclerosis, and its activation is the process of membrane translocation, that is, cPKCβII translocates from the cytoplasm to the cell membrane [20].

Oxidized low density lipoprotein (oxLDL) activates cPKCβII through binding to LOX-1 receptor of human vascular endothelial cells, promotes NK activation, p66Shc, increases the production of reactive oxygen species, then accelerates the pathogenesis of atherosclerosis and the blocker of cPKCβII can inhibit the formation of atherosclerosis [21]. In diabetic ApoE^−/−^ mice model, cPKCβ can damage vascular endothelial cells through the IL-18/IL-18 binding protein pathway, promote the formation of atherosclerosis, and cPKCβ inhibitor can relieve the progression of atherosclerosis [22]. Our group found that H_2_S level in patients with maintenance hemodialysis was lower than that in the normal population, with increased cPKCβII activation and decreased Akt phosphorylation [23]. Blocker of cPKCβII in patients with cardiovascular disease may slow the progression of cardiovascular disease [11]. These studies suggest that abnormal H_2_S metabolism was involved in the progression of uremia with cardiovascular disease and may be achieved through PKC signaling pathways.

ApoE^−/−^ mice act as one of the ideal animal models of hyperlipidemia and atherosclerosis [24]. Studies have shown that ApoE^−/−^ mice can develop atherosclerosis at 12 weeks on a high-fat diet [25]. Our previous study found that mice with ApoE^−/−^ undergoing 5/6 nephrectomy and a high-fat diet for 6 weeks can successfully establish UAAS model. Aortic atherosclerotic plaques can be present in surgery group for 6 weeks. Exogenous H_2_S donor NaHS delays formation of plaque formation in mice aorta to week 8, plaque formation was observed 4 weeks after the administration of the CSE inhibitor PPG.

In this experiment, 8-week-old male ApoE^−/−^ mice were treated with 5/6 nephrectomy and high-fat diet to make UAAS model. Mice were divided into control group, sham group, UAAS group, UAAS+L-cys group, UAAS+NaHS group, and UAAS+PPG group. We found that the activation of cPKCβII was significantly increased in UAAS group compared with control group, with decreased phosphorylation of Akt. Compared with UAAS group, the activation of cPKCβII in UAAS+L-cys group and UAAS+NaHS group was decreased, with increased phosphorylation of Akt, suggesting that exogenous H_2_S can inhibit membrane translocation of cPKCβII, thus affect the activation of cPKCβII/Akt signaling pathway. In addition, compared with UAAS group, the activation of cPKCβII was significantly increased in UAAS+PPG group, with decreased phosphorylation of Akt, suggesting that exogenous CSE inhibitors can promote the membrane translocation of cPKCβII and weaken the phosphorylation of Akt. The above results show that cPKCβII/Akt signaling pathway is involved in the formation of accelerated atherosclerosis in uremia mice mediated by an imbalance of the CSE/H_2_S system.

NO is an important gas signaling molecule [26, 27]. There are three types of nitric oxide synthase known as neuronal nitric oxide synthase (nNOS), inducible nitric oxide synthase (iNOS), endothelial nitric oxide synthase (eNOS) [28]. The production of NO in endothelial cells mainly depends on eNOS. Studies have shown that NO can inhibit platelet adhesion, leukocyte accumulation, prevent proliferation of vascular smooth muscle cells, and also participate in the occurrence and development of coronary heart disease, heart failure, atherosclerosis, and thrombosis [29, 30]. Decreased eNOS production or reduced biological activity can promote the formation of atherosclerosis by affecting the function of endothelial cells and the proliferation of smooth muscle cells [31].

H_2_S and NO have a synergistic effect on vasodilation [32]. Exogenous H_2_S can increase the relaxation of NO on blood vessels [12]. Experiments have shown that exogenous H_2_S can alleviate the inhibitory effect of OX-LDL on eNOS production, reduce oxidative stress and increase protection against vascular endothelium [33]. Exogenous H_2_S can remit the inflammation of mice ear vein endothelial cells induced by phototoxicity by increasing the synthesis of eNOS, then inhibit the formation of intravascular thrombus [34]. These studies suggest that H_2_S may regulate the production of eNOS through certain pathways, and thus affect the vascular endothelial function and participate in the formation of atherosclerosis. The activation of cPKCβII is increased in endothelial cells of patients with type 2 diabetes, and cPKCβ inhibitor LY379196 improved insulin-mediated eNOS activation. This process is involved in the occurrence of insulin resistance in diabetic patients [13]. Apigenin and naringenin can reduce the activation of cPKCβII, up-regulate the expression of eNOS and inhibit the production of oxygen free radicals, then achieve the protective effect on endothelial cells [35]. It was also shown that cPKCβII could decrease eNOS activation in the wounded tissues of diabetic mice [36]. These studies suggest that cPKCβII may participate in the regulation of vascular endothelial cell function by affecting the activity of eNOS in endothelial cells.

In this experimental study, the expression of eNOS was decreased in UAAS group compared with control group. Compared with UAAS group, the expression of eNOS was significantly increased in UAAS+L-cys group and UAAS+NaHS group, the activation of cPKCβII was decreased, and the phosphorylation of Akt was increased. This suggests that the exogenous recruitment of H_2_S donors and CSE substrates may affect the production of eNOS. In addition, compared with UAAS group, the expression of eNOS in UAAS+PPG group was decreased, the activation of membrane protein cPKCβII was increased, and the phosphorylation of Akt was decreased, suggesting that exogenous CSE synthetase inhibitors can inhibit the expression of eNOS. These results indicate that the imbalance of CSE/H_2_S system may influence the expression of downstream eNOS, regulate the production of NO, achieve the effect on the vascular endothelial inflammation, and then regulate the formation of accelerated atherosclerosis in mice with uremia. This effect may be mediated by the cPKCβII/Akt signaling pathway. Our results of this experiment showed that the imbalance of CSE/H_2_S system in mice with accelerated uremic atherosclerosis was accompanied by activation of cPKCβII/Akt signaling pathway and changes in eNOS. Suggesting that cPKCβII/Akt signaling pathway may be involved in the regulation of downstream eNOS expression mediated by the unbalanced CSE/H_2_S system in mice with accelerated atherosclerosis. However, this experiment did not provide blocker of cPKCβII/Akt signaling pathway in mice, so we cannot exclude other signaling pathways may also participate in this process. And in addition to cPKCβII/Akt signaling pathway, are there any other pathways involved, or whether there are other downstream molecules working together, these require more in-depth research in vivo and in vitro experiments.

## Conclusion

Endogenous CSE/H_2_S system may protect against the formation of UAAS via cPKCβII/Akt signal pathway. The imbalance of CSE/H_2_S system may participate in the formation of UAAS by affecting the expression of downstream molecule eNOS, which may be mediated by cPKCβII/Akt signaling pathway.

## Data Availability

All the data supporting the conclusions of this article are contained within the manuscript.

## Abbreviations

CKD: chronic kidney disease
CSE: cystathionine-γ-lyase
eNOS: endothelial nitric oxide synthase
H_2_S: hydrogen sulfide
L-cys: L-cystein
NaHS: sodium hydrosulfide group
NO: nitric oxide
PKC: protein kinase C
PPG: propargylglycine
PVDF: polyvinylidene fluoride film
UAAS: uremia accelerated atherosclerosis
